# Supplementary material for: Physical Properties and the Reconstruction of Unstable Decahedral Silver Nanoparticles Synthesized Using Plasmon-Mediated Photochemical Process
Source: Nanomaterials (Basel). 2022 Mar 24;12(7):1062. doi: 10.3390/nano12071062 (PMC9000470; doi:10.3390/nano12071062)
Supplement: Supplementary file 1 [file nanomaterials-12-01062-s001.zip › nanomaterials-1629600-supplementary.pdf]

Supporting Information

Physical Properties and the Reconstruction of Unstable Decahedral Silver Nanoparticles Synthesized Using Plasmon-Mediated Photochemical Process

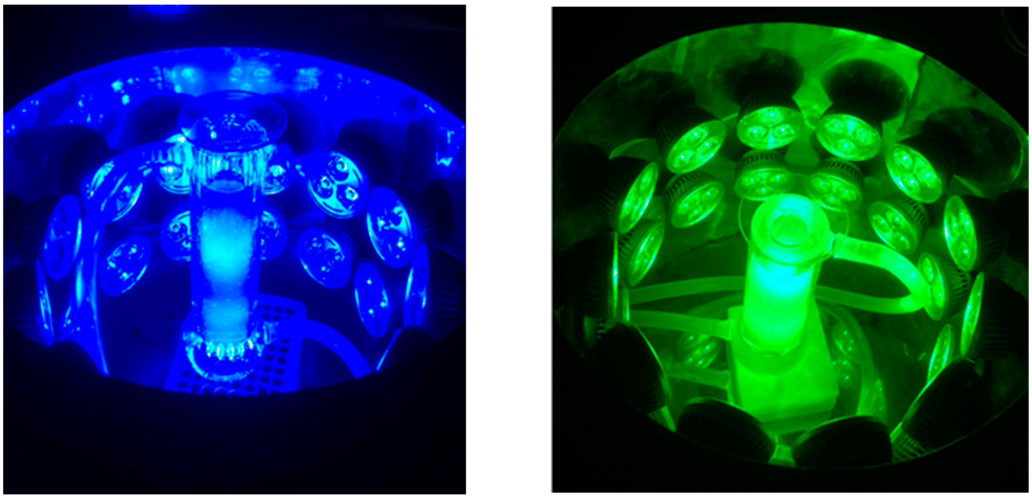

Figure S1. Setup of the temperature-controlled plasmon-mediated photochemical reaction system. The tank was connected to a circular cooling system to maintain the bath temperatures.

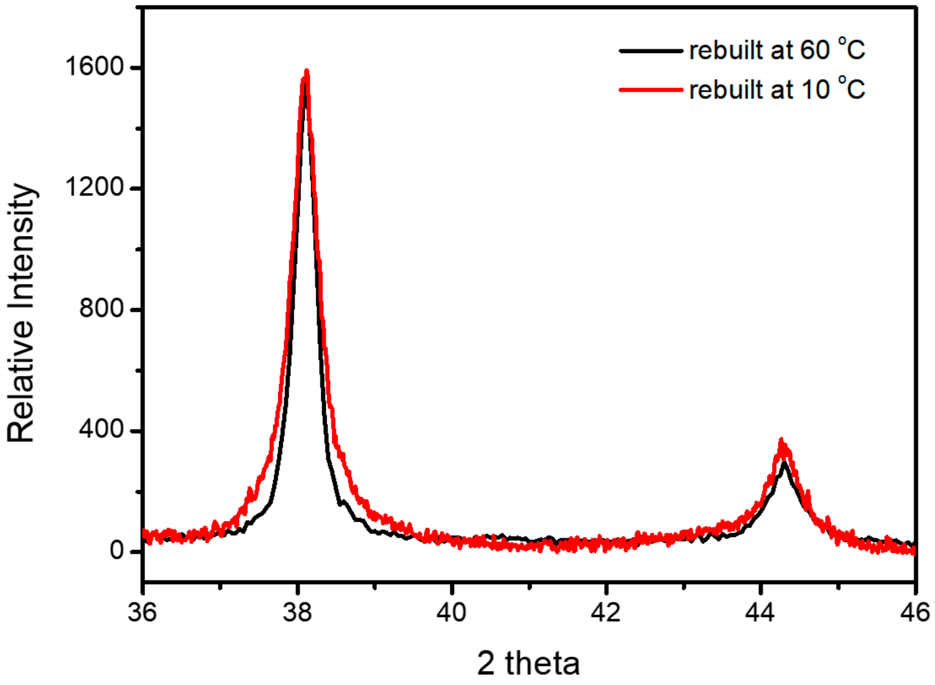

Figure S2. XRD spectra of D-AgNPs rebuilt at (A) 60 °C and (B) 10 °C, respectively.

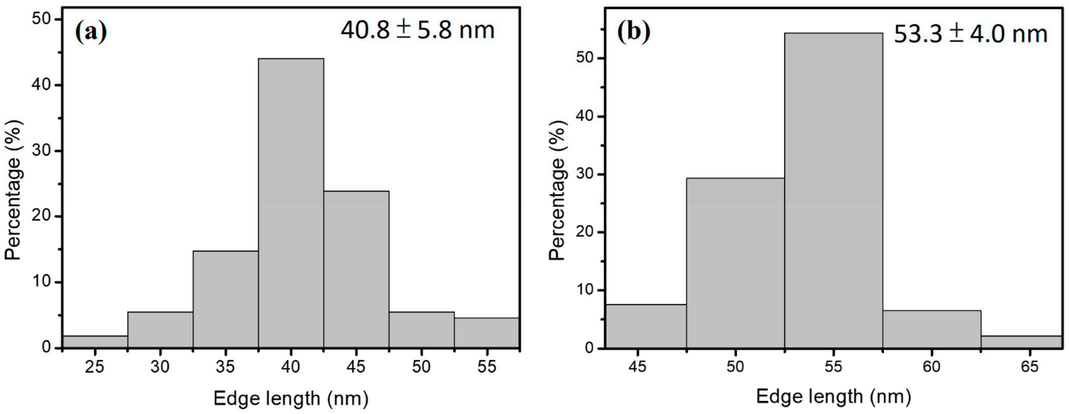

Figure S3. (**a**) Edge length distribution of D-AgNPs after the reconstruction under blue LEDs at 60 °C and (**b**) edge length distribution of D-AgNPs after the enlargement process.
